# Bile Salt Enterolith: An Unusual Etiology Mimicking Gallstone Ileus

**DOI:** 10.1155/2018/8965930

**Published:** 2018-12-19

**Authors:** Dana Ferrari-Light, Ariel Shuchleib, Joel Ricci-Gorbea

**Affiliations:** New York Presbyterian-Queens, 56-45 Main Street, Flushing NY 11355, USA

## Abstract

Primary enterolithiasis is a relatively uncommon but important cause of small bowel obstruction. We present a case of a 69-year-old male with a history of laparoscopic Roux-en-Y gastric bypass and asymptomatic duodenal diverticulum diagnosed with small bowel obstruction. CT imaging showed an obstruction distal to the jejunojejunostomy, and surgical intervention was warranted. A 4.5 cm enterolith removed from the distal jejunum was found to contain 100% bile salts, consistent with a primary enterolith. Clinicians should retain a high index of suspicion for enteroliths as a cause of small bowel obstruction, especially if multiple risk factors for enterolith formation are present.

## 1. Introduction

Enteroliths are intestinal concretions that vary widely in etiology, chemical composition, and clinical presentation. They can be categorized into primary (caused by intestinal stasis) and secondary (caused by migration via fistulous connection to the gallbladder or kidneys) [1]. Here, we present a case of primary intestinal enterolith causing small bowel obstruction that was originally diagnosed as a secondary gallstone ileus.

## 2. Case Presentation

**Figure 1:**
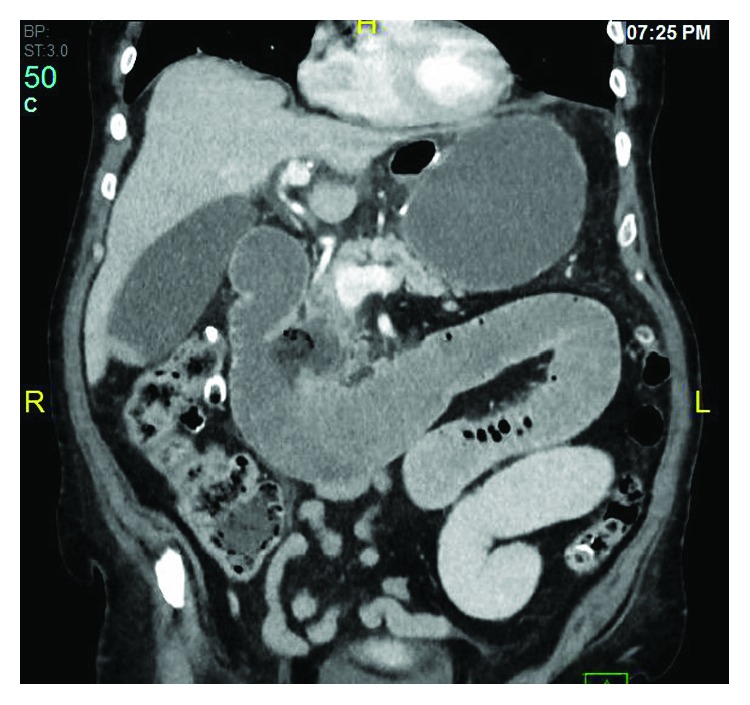
CT scan of the abdomen showing a duodenal diverticulum with dilation of the biliopancreatic limb secondary to high-grade obstruction at the distal jejunum.

**Figure 2:**
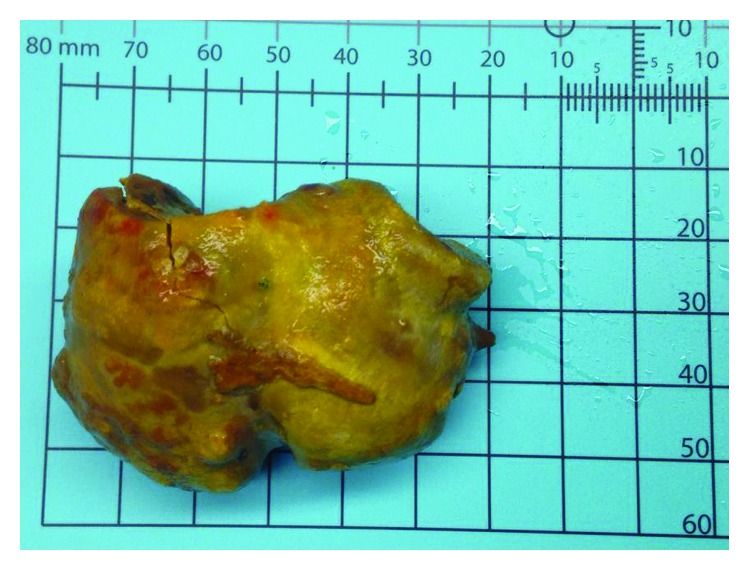
The 4.5 cm enterolith recovered from the distal jejunum/proximal ileum. Stone analysis showed this enterolith was composed of 100% bile salts.

A 69-year-old male with a remote history of laparoscopic Roux-en-Y gastric bypass 4 years earlier and a known duodenal diverticulum presented to the emergency department for bilateral upper quadrant abdominal pain for several weeks that had progressed rapidly to constant nausea, abdominal distension, and food intolerance. A CT scan of the abdomen revealed a very distended gastric remnant and what appeared to be a bowel obstruction distal to the jejunojejunostomy (Figure 1). A nasogastric tube was placed for decompression and he was taken emergently to the operating room for an exploratory laparotomy for presumed gallstone ileus. At the time of surgery, a 4.5 cm enterolith was found and removed from the distal jejunum via longitudinal enterotomy, followed by a cholecystectomy in which gallstones were found but no cholecystoenteric fistula was noted. The enterolith was sent to an outside laboratory for stone analysis and was found to contain 100% bile salts (Figure 2). The remainder of the patient's hospital stay was uneventful, and he was discharged on postoperative day 8. He remained in good health at his postoperative visit 2 weeks after surgery but then was lost to follow-up 1 month after surgery.

## 3. Discussion

This case illustrates a primary enterolith ileus that was initially thought to be a gallstone ileus. However, no evidence of a biliary-enteric fistula was found on CT scan or upon visual inspection of the gallbladder in the operating room, which further confirms the diagnosis of primary enterolith.

The diagnosis of enterolith ileus is established by observing an unremarkable gallbladder and the presence of a small bowel diverticula [2], in this case a duodenal diverticulum. Duodenal diverticulosis is a long-recognized abnormality, first described in 1710 by French physician J.B.L. Chomel [3]. It is not a particularly rare condition, affecting anywhere from 2-7% of the population [1] and is usually of little clinical significance [4]. However, it may present as diverticulitis, hemorrhage, malabsorption, or obstruction [1, 5]. When stagnation of diverticular contents is present, the rare complication of enterolith formation and obstruction may occur [2, 6, 7]. Risk factors for enterolith development include Crohn's disease, hernias, bowel diverticula, surgical anastomoses and afferent loops, small intestinal tumors, and intestinal infections including tuberculosis [2, 8–16].

Optimal management for a patient presenting with an enterolith-induced bowel obstruction includes serial abdominal examination, correction of electrolyte abnormalities, and nasogastric decompression. This noninvasive strategy may be appropriate for enteroliths less than 2 cm in diameter that are more likely to spontaneously pass through the bowel [17]. Stones larger than 2 cm should undergo surgical excision which usually includes “milking” the enterolith to a less edematous portion of the small intestine where an enterotomy is performed for removal [18–20].

Our patient exhibited several risk factors for the development of an enterolith—a preexisting duodenal diverticulum, a Roux-en-Y gastric bypass with side-to-side anastomoses and afferent loop, and a disruption of normal bowel peristalsis secondary to his bariatric surgery [8]. We believe that these conditions promoted precipitation of bile salts in his duodenal diverticulum, allowing a large enterolith to form. These alterations in intestinal anatomy and function are key to making the diagnosis of bile salt enterolith obstruction in this patient, and a detailed history and physical exam are paramount to this investigation. Radiological studies including upper gastrointestinal series or CT scans should be used to investigate possible sources of bowel obstruction and to exclude mimics. Surgical management should be centered around obstruction relief and prevention of recurrence. Improved diagnostic imaging and innovations in laparoscopic and minimally invasive surgery will provide patients with decreased morbidity versus traditional laparotomy procedures.
